# A Case Report: Lisdexamfetamine-Induced Delusional Parasitosis

**DOI:** 10.7759/cureus.81305

**Published:** 2025-03-27

**Authors:** Xialing Ann Chen, John Kelsey

**Affiliations:** 1 Psychiatry, University of California San Francisco, San Francisco, USA; 2 Psychiatry, Sutter Health California Pacific Medical Center, San Francisco, USA

**Keywords:** attention-deficit hyperactivity disorder (adhd), clinical psychiatry, clinical psychiatry neurology, delusional parasitosis, dermatology and psychiatry, drug-induced psychosis, ekbom syndrome, lisdexamfetamine, stimulant treatment, vyvanse

## Abstract

Attention-deficit hyperactivity disorder (ADHD) is generally treated with stimulant medications without significant complications. Delusional parasitosis (Ekbom syndrome) can occur secondary to ADHD treatment. It is a rare condition defined as having a fixed, false belief that one is infected with insects, parasites, or organisms and that one experiences cutaneous sensations without any clinical evidence of infestation. Although stimulant treatment with methylphenidate or mixed amphetamine salts has been associated with delusional parasitosis, there is yet a case in the literature illustrating delusional infestation secondary to lisdexamfetamine. The following case is unique in that lisdexamfetamine caused delusional parasitosis in a 53-year-old man with ADHD who previously tolerated mixed amphetamine salts and armodafinil without side effects. The discontinuation of lisdexamfetamine, coupled with a second-generation antipsychotic, quickly resolved the delusion. For those who may prescribe lisdexamfetamine or treat patients with ADHD, it is crucial to carefully assess medication use, as discontinuation or dose adjustment of the suspected causative drug can have a positive impact on the course of delusional parasitosis.

## Introduction

Attention-deficit hyperactivity disorder (ADHD) is one of the most common neuropsychiatric disorders in children and adolescents and usually continues into adulthood [1]. The overall prevalence of current adult ADHD is 4.4%, with higher prevalence in men (5.4%) as opposed to women (3.2%), and non-Hispanic Caucasians have a higher prevalence than any other race or ethnicity [2]. ADHD symptoms include impulsivity, hyperactivity, inattention, executive function impairment, and emotional dysregulation resulting in functional impairment [3]. The pathogenesis is unknown. Some structural brain abnormalities related to ADHD functional impairments can be seen on CT and MRI: smaller volumes in the frontal cortex, cerebellum, and subcortical structures and abnormalities in the prefrontal and parietal circuits [3]. Given the catecholaminergic nature of many brain circuits, hypoactivity of dopamine and norepinephrine in the frontal-subcortical circuits likely underlie the brain and functional impairment in ADHD [4]. Therefore, treatment involves stimulant medications such as amphetamines (dextroamphetamine or lisdexamfetamine) or methylphenidate. They block the dopamine transporter or the norepinephrine transporter, which will increase free brain levels of noradrenaline and dopamine, effectively reducing ADHD symptoms [5].

Stimulant medications are generally carefully titrated and well-tolerated when taken as directed. Common side effects in adults treated for ADHD include insomnia, dry mouth, decreased appetite, irritability, headache, or dysphoria [6]. Treatment emergent psychotic or manic symptoms in patients without prior history was noted to occur at a rate of 0.1% (four patients with events out of 3482 exposed to methylphenidate or amphetamine for several weeks at usual doses) of stimulant-treated patients compared to zero in placebo-treated patients in a pooled analysis of multiple short-term, placebo-controlled studies [7]. Although psychosis is an unusual side effect, delusional parasitosis (Ekbom syndrome) can occur secondary to ADHD treatment with stimulants [7].

Delusional parasitosis is a rare condition defined as having a fixed, false belief that one is infected with insects, parasites, or organisms and experiencing cutaneous sensations without any clinical evidence of infestation [8]. Patients become preoccupied with the idea of being infested that they interpret normal skin markings such as old scars or skin pigmentation as evidence, and some compulsively bring “evidence” to display to medical professionals known as the “baggie sign,” "matchbox sign," "Ziploc bag sign," or "specimen sign" [8]. Others might have tactile and visual hallucinations of formication, defined as a sensation of insects or parasites crawling on or burrowing into the skin, resulting in self-inflicted excoriations, distress, and potential skin infection [9]. Patients often avoid psychiatrists and present to the emergency department, urgent care, or primary care seeking medical treatment for perceived parasites [8]. Etiology can be primary, such as due to schizophrenia, or secondary, such as due to medical problems, substance use, or prescription drugs [10]. The pathogenesis of delusional parasitosis is unknown but theorized to involve dysregulation of striatal dopamine systems, which are normally responsible for regulating attention, reward, and motor coordination [11]. According to the Diagnostic and Statistical Manual of Mental Disorders, Fifth Edition, Text Revision (DSM-5-TR), delusional parasitosis (classified as Delusional Disorder, Somatic Type) is diagnosed by the presence of one or more persistent delusions lasting at least one month specifically involving the false belief of infestation by parasites or insects, without meeting schizophrenia criteria, functioning otherwise relatively intact apart from the delusional belief, and symptoms not attributable to substance use, medical conditions, or other mental disorders [8].

Although stimulant treatment with methylphenidate, atomoxetine, or extended-release mixed amphetamine salts has been reported to cause delusional parasitosis, there is yet a case in the literature illustrating delusional infestation secondary to lisdexamfetamine [9,12]. In the blood, lisdexamfetamine is converted to dextroamphetamine, a noncatecholamine sympathomimetic amine with central nervous system (CNS) stimulant activity [13]. In the CNS, amphetamines block the reuptake of catecholamines norepinephrine and dopamine into the presynaptic neuron, resulting in the increased release of these monoamines into the extraneuronal space [13]. The mode of therapeutic action of lisdexamfetamine in the treatment of ADHD is not known [13]. Per FDA drug warnings and precautions, lisdexamfetamine may cause new-onset hallucinations, delusions, or mania at recommended doses, even in patients without prior psychiatric diagnosis, but there are limited reports of such side effects in the literature and likely none of delusional parasitosis secondary to lisdexamfetamine [13]. The following case is unique in that lisdexamfetamine caused delusional parasitosis in a 53-year-old man with ADHD who previously tolerated mixed amphetamine salts and armodafinil without side effects.

## Case presentation

The patient was a 53-year-old man with a history of depression, anxiety, heterozygous MTHFR mutation, type 1 diabetes on insulin, sleep apnea, and mild cognitive impairment who presented to the outpatient psychiatry clinic for depression and ADHD. The patient endorsed symptoms of trouble concentrating, difficulty understanding what he read, and trouble remembering deadlines. He was prescribed armodafinil for low energy associated with sleep apnea, and he tolerated it well at 250 mg daily. He was previously prescribed dextroamphetamine-amphetamine up to 30 mg daily for ADHD, without significant side effects, but it was stopped due to a lack of sustained efficacy despite an initial robust benefit. He requested to switch from armodafinil to lisdexamfetamine to help with concentration and focus. Armodafinil was slowly downtitrated as lisdexamfetamine was started at 30 mg qAM daily. At lisdexamfetamine 30 mg daily, the patient endorsed improved clarity and denied mania, psychotic symptoms, or other side effects. It was then increased to 50 mg daily, which he tolerated well. Armodafinil was stopped, while he continued to take lisdexamfetamine 50 mg daily for ADHD, trazodone 25 mg qhs for insomnia, as well as bupropion XL 450 mg and sertraline 125 mg for depression. Other medications included ascorbic acid, atorvastatin, vardenafil, diclofenac, ergocalciferol, ferrous gluconate, fluticasone propionate, lisinopril, naloxegol oxalate, tadalafil, tapentadol, valacyclovir, and tretinoin. No other herbal or over-the-counter medications were reported.

After lisdexamfetamine was titrated to 70 mg daily, about six weeks later, the patient reported improvement in energy, memory, and attention; however, he started having delusional parasitosis signs and symptoms. He presented to the outpatient dermatology clinic with a reported rash all over his body, predominantly on his arms and hands, and stated that he saw bugs and eggs. He used a handheld magnifier to visualize and demonstrate his findings. Per dermatology note, the patient declined a total body skin exam; only areas demonstrated by the patient were examined. Numerous erosions were noted on bilateral upper arms and the lateral aspect of the upper back, many with hemorrhagic crust but no burrows. Patient perseverated on infestation, insisting that he could see the organisms, to which the dermatologist diagnosed him with delusional parasitosis. The patient next presented to urgent care with similar complaints, and the urgent care provider prescribed permethrin for presumed scabies. He then went to see his primary care physician for black spots on his palm and skin pruritus, and he brought a plastic bag with picked skin samples inside to be sent to pathology. Path result showed fragments of parakeratotic stratum corneum with no scabies, parasites, or fungal organisms. He also complained of diarrhea due to parasites in the stool. Ova and parasite were negative; *Giardia *and *Cryptosporidium *Enzyme Immunoassay (EIA) tests were negative; stool culture was negative; leukocyte fecal smear was negative; treponema pallidum Ab by TP-PA was negative; basic metabolic panel (BMP) showed glucose 199; complete blood count (CBC) was unremarkable; HIV was negative; and thyroid-stimulating hormone (TSH), folate, and B12 were normal. He then went to the emergency room for parasitic infestation, showing videos on his iPhone and stating that his roommate could confirm, but he was sent home as his skin lesions were inconsistent with parasitosis. He visited another dermatologist for a second opinion, because he felt that the first dermatologist did not do a microscopy exam and that the pathology result was a “mindfuck” per his review of online medical resources.

When the patient returned to see his outpatient psychiatrist, his outpatient psychiatrist suspected that his delusional parasitosis was secondary to lisdexamfetamine, though it was unclear if the patient took extra pills, which caused the delusion, or if it was an underlying side effect of the drug. The patient denied that he had recent medication changes, denied taking higher than 70 mg daily of lisdexamfetamine, and denied using any illicit substances. The patient had no history of recreational drug use. Urine toxicology tests were unremarkable. Lisdexamfetamine was stopped. He was prescribed aripiprazole 5 mg daily, given that he previously refused risperidone. Once lisdexamfetamine was discontinued, the patient’s delusional parasitosis resolved. He no longer believed that he had bugs: “I don’t know what that all was. My skin has cleared.” Aripiprazole was discontinued. The patient requested to be restarted on armodafinil for energy and mood, which he tolerated well without side effects. Lisdexamfetamine was added to his drug allergy list. His delusional parasitosis did not return at his subsequent monthly visits.

## Discussion

Delusional parasitosis can be secondary to certain drugs, with anti-Parkinson’s drugs being most frequently associated, followed by antidepressants, antiepileptics, antibiotics, and prescription stimulants [14]. There was a case report of an individual with ADHD who was treated with atomoxetine who developed delusional parasitosis that resolved with the cessation of the drug and initiation of antipsychotic medications [9]. There was another case of a woman with ADHD treated with extended-release mixed amphetamine salts, resulting in delusional parasitosis that resolved once the drug was discontinued [12]. There have been numerous reports of psychosis and delusions secondary to stimulant treatment, including methylphenidate [9]. However, this case is unique in that the patient tolerated amphetamine/dextroamphetamine salts and armodafinil without complications, yet developed delusional parasitosis with lisdexamfetamine. Given the temporal relationship between the onset of the symptoms and the prescription of lisdexamfetamine, as well as the rapid and complete resolution after discontinuation of lisdexamfetamine and administration of an antipsychotic medication, lisdexamfetamine was the likely cause. To the best of our knowledge, there is no other case report of delusional infestation secondary to lisdexamfetamine treatment in the current literature. It is important for those who may prescribe this drug or treat patients with ADHD to be aware of this side effect. This is true since prompt discontinuation or dose adjustment of the suspected drug may have a positive influence on the course of delusional parasitosis.

The mechanism of action behind drug-induced delusional parasitosis likely stemmed from alterations in neurotransmitters, predominantly dopamine, in the CNS. Consistent with the well-known theories regarding the level of dopamine in the CNS as a major factor contributing to delusional parasitosis and other delusional disorders, the drugs associated with delusional parasitosis have dopaminergic activities either as a major mechanism or a side mechanism [14]. The main etiological foundation of this hypothesis was the decreased functioning of the striatal dopamine transporter that was a key regulator of the dopamine-reuptake system, resulting in higher levels of dopamine in the striatum, therefore hyperactivity of dopaminergic neurons and thus formation of delusions [14]. Thus, treatment of delusional parasitosis involves drugs with antidopaminergic potency. Pimozide was historically the first-line treatment, but due to possible QTc prolongation and extrapyramidal side effects, second-generation antipsychotics have been preferred with good efficacy and side-effect profile, especially risperidone [11]. Aripiprazole is a good second option [11].

In addition to using antipsychotics to reduce the effect of extracellular dopamine via postsynaptic D2 receptor blockade in the treatment of delusional parasitosis, it is important to assess toxicological screenings to rule out misuse of substances in patients presenting with new-onset delusions. Amphetamine and cocaine can cause delusion or psychosis via activation of the mesolimbic dopamine pathway, while withdrawal from heroin or alcohol can cause formication [14]. Medication use and substance misuse should be carefully assessed to properly diagnose and treat delusional parasitosis.

## Conclusions

For patients presenting with new-onset delusional parasitosis, it is important to assess the patient’s medication list as stimulant treatment for ADHD can cause delusions, mania, or other psychotic symptoms even in patients without prior psychiatric history. It is also crucial to obtain a toxicology screen, as illicit drug use or withdrawal from certain substances can cause delusional parasitosis. Prompt recognition and discontinuation of the putative causative agent can have a significant, positive effect on the treatment of delusional parasitosis. Antipsychotic medications, such as risperidone, should be started to treat the delusion. It is also essential to build a therapeutic alliance and avoid direct confrontation about the existence of parasites or organisms in patients presenting with delusional parasitosis because if patients trust the physicians, they are more likely to adhere to antipsychotic treatment and refrain from misusing the causative drug. Since physicians other than psychiatrists tend to be the first point of contact for patients presenting with delusional parasitosis, recognizing the signs and symptoms of delusional parasitosis, such as the “baggie sign,” is paramount, especially since patients tend to avoid psychiatrists for such complaints. However, involving psychiatry as soon as possible might help decrease the frequency of patients presenting to primary care, urgent care, emergency department, or dermatology for parasitic skin lesions. Not only will that decrease the burden on the healthcare system, but it will also lead to prompt and proper diagnosis and treatment, alleviating patient suffering and ensuring better quality of life.
